# Vitamin D Protects against Traumatic Brain Injury via Modulating TLR4/MyD88/NF-*κ*B Pathway-Mediated Microglial Polarization and Neuroinflammation

**DOI:** 10.1155/2022/3363036

**Published:** 2022-07-15

**Authors:** Hongsheng Jiang, Xinyu Yang, Yanzhou Wang, Caifeng Zhou

**Affiliations:** ^1^Department of Neurosurgery, Cangzhou Central Hospital, Cangzhou, China; ^2^Department of Neurosurgery, Tianjin Medical University General Hospital, Tianjin, China

## Abstract

Vitamin D (VD) deficiency is associated with neuroinflammation and neurocognitive deficits in patients with traumatic brain injury (TBI). The present study was aimed at investigating the therapeutic effects of VD and the molecular mechanisms after TBI. After the intraperitoneal injection of VD (1 *μ*g/kg), sensorimotor and cognitive function was assessed via a series of behavioral tests in TBI rats. Traumatic outcomes were investigated by brain edema, blood-brain barrier (BBB) disruption, and morphologic staining. In vitro, cellular viability and cytotoxicity in primary hippocampal neurons were detected via the MTT method and LDH release. Hippocampal oxidative stress-related enzymes and proinflammatory mediators and the serum concentration of VD were analyzed by ELISA. The expression of VDR, TLR4, MyD88, and NF-*κ*B p65 was measured by Western blot. Furthermore, the levels of M1/M2 microglial markers were quantified using real-time PCR and Western blot. VD treatment significantly increased the serum level of VD and the hippocampal expression of VDR. VD not only effectively alleviated neurocognitive deficits, brain edema, and BBB disruption but also promoted hippocampal neuronal survival in vivo and in vitro. Moreover, VD therapy prevented excessive neuroinflammation and oxidative stress caused by TBI. Mechanically, the hippocampal expression of TLR4, MyD88, and nuclear NF-*κ*B p65 was elevated in the TBI group but robustly restrained by VD treatment. Taken together, VD provides an important neuroprotection through modulating hippocampal microglial M2 polarization and neuroinflammation via the TLR4/MyD88/NF-*κ*B pathway.

## 1. Introduction

Vitamin D (VD), a steroid hormone, is well recognized as a neurosteroid that modulates brain development, maintains adult brain function, and delays brain aging [1]. 1,25-Ddihydroxy-vitamin D (1,25(OH)2D3) is the active form of VD and participates in the regulation of calcium and phosphate metabolism [2]. Recently, accumulated data has unraveled that active VD not only had a pivotal role in proliferation, differentiation, immunological modulation, and gene transcription but also contributed to neuroprotection, neurotrophism, neurotransmission, synaptic plasticity, and neural circuit connectivity [3]. VD has the genomic and nongenomic actions in the brain. The transcriptional activity of VD is mediated via the nuclear VD receptor (VDR) [4]. VDR, a member of the nuclear receptor family, are widely expressed in all major cell types of the embryonic and adult brain, including neurons and glial cells in multiple brain regions [5]. The immunoreactivity of VDR was noted to be strongest in CA1 and CA2 pyramidal cells in the human hippocampus [6]. Of note, VDR contains a highly conserved DNA-binding domain and a ligand-binding domain; after ligand binding to VD, VDR forms a heterodimer with the retinoic acid X receptor (RXR) [7]. The VDR/RXR complex binds to VD response elements (VDREs) in the promoter region, introns, and intergenic sites of target genes [8]. The VD/VDR/RXR/VDRE complex may enhance transforming growth factor-*β* (TGF-*β*) transcription and inhibit the transcription of parathyroid hormone and 1-*α*-hydroxylase [9]. The nongenomic actions of active VD are mediated by binding a specific plasma membrane receptor. Interestingly, an increasing amount of epidemiological studies have pointed to the link between VD deficiency and an increased risk of neurological and psychiatric diseases including Alzheimer's disease (AD), Parkinson's disease (PD), autism, depression, and schizophrenia [10–12]. VD treatment was reported to act as an anti-inflammatory effect in a genetic model of age-related glaucomatous neurodegeneration (DBA/2J mice) via reducing microglial and astrocyte activation and inhibiting pNF-*κ*B-p65 and inflammatory cytokine expression [13]. In asthmatic mice, VD administration restored the Th17/Treg balance and airway inflammation by suppressing the NF-*κ*B pathway [14].

Traumatic brain injury (TBI) is the leading cause of death and disability worldwide. The primary acute event is caused by mechanical damage during initial impact, and secondary brain injury results from delayed intracellular signaling pathways and neurochemical process, such as calcium overload, glutamatergic excitotoxicity, oxidative stress, and inflammatory response [15]. After TBI, microglia and astrocytes release proinflammatory cytokines, such as tumor necrosis factor-*α* (TNF-*α*), interleukin- (IL-) 1*β*, and IL-6, leading to a local neuroinflammatory response, and these inflammatory mediators might mobilize immune cells to the injury regions, resulting in further neuroinflammatory reactions [16]. Posttraumatic neuroinflammation has been reported to contribute to the widespread tissue damage, neuronal loss, and neurological dysfunctions, leading to secondary brain injury following TBI. Neuroinflammation is characterized by increased proinflammatory cytokine production, microglial activation, and enhanced BBB permeability [17]. Noticeably, numerous clinical studies have highlighted that VD supplementation improved consciousness status and inflammatory biomarkers [18], promoted long-term performance and cognitive function recovery [19, 20], and improved quality of life in patients with TBI [21]. Additionally, a preclinical research has indicated that VD therapy alleviated BBB disruption and cognitive dysfunction in the TBI rat model [22]. Although VD exhibits a favorable effect on TBI outcomes, the precise molecular mechanism underlying these neuroprotective effects is poorly understood.

In this study, we attempt to explore whether VD therapy plays a central role in neurocognitive dysfunction, neuronal survival, oxidative stress, and neuroinflammation and further elaborate the potential molecular signaling mechanism in a rat model of TBI.

## 2. Material and Methods

### 2.1. Animals

A total of 220 male Sprague-Dawley (SD) rats (age, 12–16 weeks; weighing 280–320 g) were purchased in Beijing HFK Biotechnology Co., Ltd. (Beijing, China). All experimental protocols were approved by the Animal Ethics Committee of Tianjin Medical University. All rats in this study were housed in a standard temperature room (21-24°C, 30-70% humidity) and maintained on a 12 h light/dark cycle with food and water available ad libitum. The animals were acclimated for at least 1 week before surgery.

### 2.2. Controlled Cortical Impact Model and Drug Treatment

The rat TBI model was induced by controlled cortical impact (CCI) to cause brain trauma, in accordance with the study previously described by Dixon et al. [23]. Briefly, SD rats were anesthetized by intraperitoneal injection of pentobarbital sodium (60 mg/kg). Precise and repeatable brain injury was performed using a commercial CCI device (Leica Microsystems). The parameters were as follows: velocity, 6.0 m/sec; impact depth, 5.0 mm; and contact time, 50 msec. After TBI induction, the rats presented with hair standing up, urinary incontinence, limb convulsions, and other manifestations, and some rats had temporary paralysis and partially visible dilated pupils on one or both sides. In the sham group, rats underwent anesthesia and the same procedure except for the CCI injury. After induction, rats were intraperitoneally injected with VD (1 *μ*g/kg) at 30 min, 12 h, and 24 h post-CCI in the TBI+VD group.

### 2.3. Primary Cultures of Hippocampal Neurons and Treatment

Hippocampal neurons were obtained from newborn SD rats, and the brains were collected in Hanks' balanced salt solution (HBSS), the cerebral cortex and the meninges were removed, and the hippocampus was dissected and cut into small pieces. Hippocampal tissues were digested in 0.25% trypsin (Gibco, MA, USA) at 37°C for 20 min and were resuspended with DMEM supplemented with 10% fetal bovine serum (Gibco, MA, USA). Then, the cells were plated on coverslips coated with poly-L-lysine (0.1 mg/ml, Sigma, St. Louis, MO, USA) at a density of 100,000/well. After overnight incubation in a 37°C, 5% CO_2_ incubator, the tissue was washed and exchanged with a defined medium: Neurobasal-A medium containing 2% B-27, 0.5 mM glutamine, 25 mM glutamate, and 1% penicillin/streptomycin (both from Gibco, MA, USA). After 7 days of culture, hippocampal neurons were identified by immunofluorescent staining of microtubule-associated protein-2 (MAP-2) protein. Primary neurons were incubated with LPS (1 *μ*g/ml) for 6 h to induce neuron injury in vitro.

### 2.4. Evaluation of Cellular Viability and Cytotoxicity

Cell viability was assessed using the dye 3-(4, 5-dimethyl thiazol-2-yl)-2, 5-diphenyltetrazolium bromide (MTT, Beyotime, Beijing, China) method. 20 *μ*l MTT solution (5 mg/ml) was added to each well (1 × 10^3^ cells/well) and incubated for 2 h at 37°C. Afterward, the supernatant was removed followed by the addition of 150 *μ*l DMSO to dissolve the formazan crystals. The absorbance at 570 nm was monitored with a microplate reader (Thermo Scientific, USA). Cytotoxicity was estimated by quantifying the cytoplasmic enzyme lactate dehydrogenase (LDH) released by damaged neuron into the culture medium using the LDH Cytotoxicity Assay Kit (Beyotime, Beijing, China) according to the manufacturer's instructions.

### 2.5. Neurological Severity Score

Posttraumatic sensorimotor impairments were evaluated using the modified Neurological Severity Score (mNSS) test. The test was carried out on all rats at 1 d, 3 d, 7 d, and 14 d post-TBI. Five rats in each group were used for the mNSS test. The mNSS was graded using a scale of 1 to 18. The mNSS includes a composite of sensory, motor, balance, and reflex tests in rats after TBI.

### 2.6. Rotarod Test

The rotarod test was performed to assess motor coordination at 1 d, 3 d, 7 d, and 14 d post-TBI in rats after the mNSS test. Briefly, the rats were placed on a rotating rod, which accelerated from 4 rpm and increased gradually to 40 rpm within 300 s. During the procedure, the latency to fall off the rotating rod was recorded as the time by blinded experimenters, and the average latency from three trials was used for analyses.

### 2.7. Morris Water Maze Test

The Morris water maze (MWM) test was utilized to measure posttraumatic spatial learning and memory at 9-14 d after TBI in rats. Five rats in each group were tested with 4 trials per day for 5 consecutive days from days 9-13 after surgery. Briefly, animals were randomly placed into a quadrant and allowed a maximum of 60 sec to escape to the platform. Animals that failed to find the platform within 90 sec were placed on the platform for 10 sec. During this process, the latency to escape to the hidden platform was recorded and analyzed to assess spatial learning ability following injury. After trials, the hidden platform was removed from the quadrant, and the probe test was conducted at 14 d postinjury. In the probe trial, time spent in the target quadrant and the platform crossing time were recorded to evaluate the spatial memory capacity.

### 2.8. Assessment of Brain Edema and Blood-Brain Barrier Disruption

Brain water content (BWC) was analyzed using the wet weight-dry weight method at 1 d, 3 d, 7 d, and 14 d posttrauma. There were 5 rats in each group at each time point. Briefly, brain tissues from the anesthetized rats were separated and weighed to obtain wet weight. After drying the samples at 100°C for 24 h, dry weight was determined. BWC was calculated using the following formula: BWC (%) = [(wet weight − dry weight)/wet weight] × 100%. The expression level of albumin was quantified using the Western blot assay to evaluate the disruption of BBB function at 3 d post-TBI. Five rats in each group were used for the measurement of BBB permeability.

### 2.9. H&E Staining and Nissl Staining

Five rats in each group were employed for morphologic observation at 3 d posttrauma. After anesthesia, brain tissues were quickly extracted and fixed in 4% paraformaldehyde for 48 h-72 h, embedded in paraffin, and cut into 5 *μ*m coronal sections for H&E staining and Nissl staining. Then, the sections were stained with hematoxylin and eosin (H&E) solution. The sections were stained with a Nissl staining solution at 57°C for 60 min. After washing, the samples were differentiated in a Nissl differentiation solution at room temperature.

### 2.10. Enzyme-Linked Immunosorbent Assay (ELISA)

The concentrations of proinflammatory cytokines were detected in the hippocampal tissues at 1 d, 3 d, 7 d, and 14 d post-CCI. There were 5 rats in each group at each time point for ELISA analysis and oxidative stress assessment. Hippocampal supernatants were collected to measure the contents of proinflammatory cytokines, such as TNF-*α*, IL-1*β*, and IL-6 using the ELISA kits according to the manufacturer's protocols (Anoric-Bio, Tianjin, China). Additionally, the serum concentration of 25(OH)D3 also was detected by ELISA at 1 d, 3 d, 7 d, and 14 d postinjury.

### 2.11. Measurement of SOD, GSH-PX, and MDA

The levels of superoxide dismutase (SOD), glutathione peroxidase (GSH-PX), and malondialdehyde (MDA) were detected to assess the effect of VD treatment on oxidative stress after TBI at 1 d, 3 d, 7 d, and 14 d post-CCI. Among them, MDA is the measure of lipid peroxidation. Briefly, rat hippocampal tissues were removed, homogenized, and centrifuged at 2000 g for 15 min. The activities of SOD, GSH-PX, and MDA in the supernatant were determined using commercial kits (Nanjing Jiancheng Bioengineering Institute, Nanjing, China) according to the instruction of the manufacturer. And the final units are represented as nmol/mg protein.

### 2.12. Real-Time PCR

RNA samples from the hippocampal tissues at 1 d, 3 d, 7 d, and 14 d post-TBI were prepared using TRIzol (Invitrogen, Carlsbad, CA, USA) according to the manufacturer's instructions. Five rats in each group were employed for real-time PCR analysis. RNA (2 *μ*g) was employed for the synthesis of complementary DNA (cDNA) using the PrimeScript RT kit (DRR047A, Takara, Japan). Real-time PCR was performed using the TaqMan system (Applied Biosystems) in a total volume of 20 *μ*l with cDNA, primers, and 40 cycles of PCR. The 2^−∆∆*Ct*^ method was used to calculate the relative level of gene expression using GAPDH as a reference gene.

### 2.13. Western Blot Analysis

Total proteins were isolated from the hippocampal tissues 3 d post-TBI using RIPA protein lysate (Beyotime, Shanghai, China) and quantified using the bicinchoninic acid (BCA) assay kit (Beyotime, Shanghai, China). Five rats in each group were employed for Western blot analysis. The protein was separated by 10% polyacrylamide-SDS gels and transferred onto PVDF membranes. The membranes were blocked with 5% nonfat dry milk at 4°C overnight and then incubated with primary antibodies (1 : 1000, Santa Cruz, CA, USA) at 4°C overnight. Next, the membranes were incubated with a horseradish peroxidase- (HRP-) conjugated secondary antibody (1 : 5000, ABclonal, Wuhan, China) at room temperature for 2 h. Finally, the blots were developed using Peirce ECL Western Blotting Substrate (Thermo Fisher Scientific, USA). The protein expressions were normalized to *β*-actin expression.

### 2.14. Statistical Analysis

Data were analyzed using SPSS version 17.0 software, and SigmaPlot software (V12.5, USA) was used for the creation of the figures. All data were presented as the means ± standard deviation (SD). Multiple group means were compared by one-way analysis of variance (ANOVA) with the post hoc Bonferroni correction. *P* value < 0.05 was considered statistically significant.

## 3. Results

### 3.1. Vitamin D Supplement Increased the Serum Concentration of VD and the Hippocampal Expression of VDR

**Figure 1:**
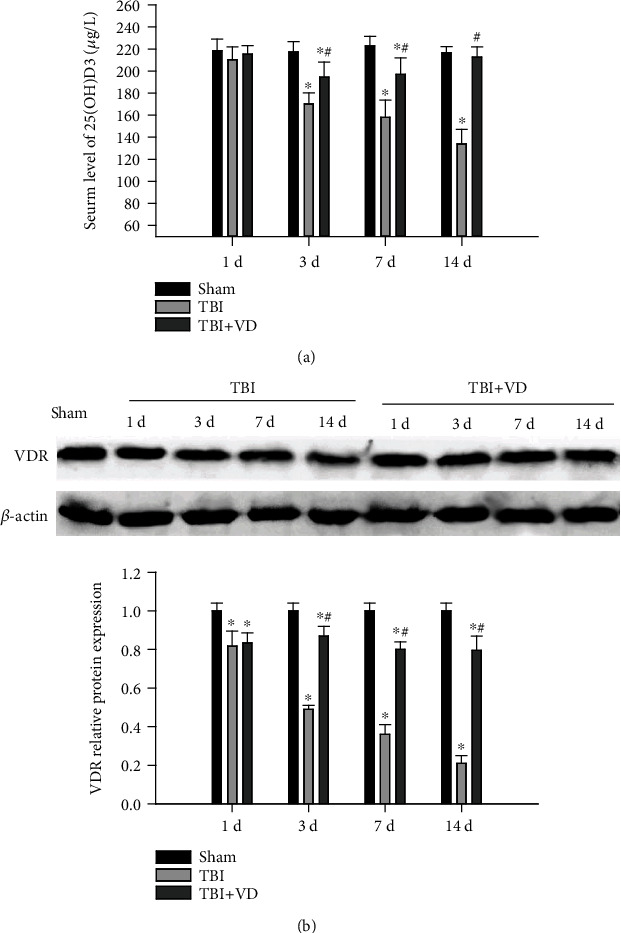
The effects of VD on the serum concentration of VD and VDR expression in the hippocampus. (a) The serum concentration of VD was determined using ELISA analysis at 1 d, 3 d, 7 d, and 14 d post-TBI. (b) The hippocampal expression of VDR protein was quantified by Western blot at 3 d post-TBI in rats. The data are represented as mean ± SD from three independent experiments. ^∗^*P* < 0.05 compared with the sham group, ^#^*P* < 0.05 compared with the TBI group.

VD content might be assessed by serum concentration of 25(OH)D3 that is the main circulating form of VD in the body, with a half-life of 2-3 weeks [24]. After VD administration, the serum concentration of 25(OH)D3 was measured via ELISA analysis. Results indicated that 25(OH)D3 content in the serum was significantly lower in the TBI group than in the sham group (*P* < 0.05), indicating that VD deficiency occurs in the rat model of TBI. Compared with the TBI group, the serum concentration of 25(OH)D3 was significantly increased in the TBI+VD group at 3 d, 7 d, and 14 d post-TBI (*P* < 0.05), suggesting that VD treatment could ameliorate CCI-induced VD deficiency in rats (Figure 1(a)). We also quantified VDR protein expression in the hippocampal tissue of rat via Western blot. The hippocampal expression of VDR protein was evidently reduced in the TBI group compared to the sham group at 1 d, 3 d, 7 d, and 14 d post-TBI (*P* < 0.05). Compared with the TBI group, VD significantly enhanced the expression of VDR in the damaged hippocampus post-TBI (*P* < 0.05, Figure 1(b)).

### 3.2. Vitamin D Promoted Neurological Function Recovery after TBI

**Figure 2:**
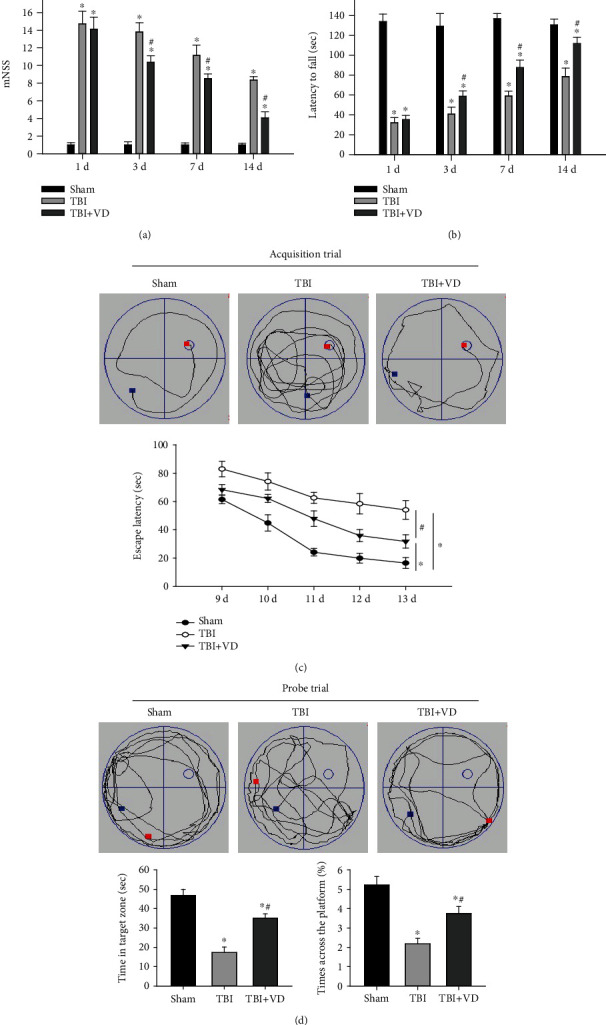
The effects of VD on neurological and cognitive functions in rats. (a) Neurological severity was assessed using mNSS at 1 d, 3 d, 7 d, and 14 d posttrauma in rats. (b) Motor coordination was monitored via the rotarod test at 1 d, 3 d, 7 d, and 14 d posttrauma. (c, d) Swimming paths, escape latency, time spent in the target zone, and times across the platform in the Morris water maze test presented the changes of spatial learning and memory ability. The data are represented as mean ± SD from three independent experiments. ^∗^*P* < 0.05 compared with the sham group, ^#^*P* < 0.05 compared with the TBI group.

We first assessed the impact of VD on neurological deficits following TBI by the mNSS and the rotarod test. Rats in the TBI group had significantly higher mNSS and shorter duration to stay on a rotating rod than those in the sham group at 3 d, 7 d, and 14 d post-TBI (*P* < 0.05, Figures 2(a) and 2(b)). Compared with the TBI group, rats in the TBI+VD showed apparently improved sensorimotor performance. In addition, the MWM test was performed to estimate spatial learning and memory function with the acquisition trial (from 7 d to 11 d postinjury) and the probe trial (12 d postinjury). The results displayed that VD conspicuously ameliorated posttraumatic cognitive impairments as evidenced by the decreased escape latency to the hidden platform (*P* < 0.05, Figure 2(c)), the increased time spent in the target zone (*P* < 0.05), and times across the platform compared with the TBI group (*P* < 0.05, Figure 2(d)).

### 3.3. Vitamin D Reduced Secondary Brain Injury Induced by TBI

**Figure 3:**
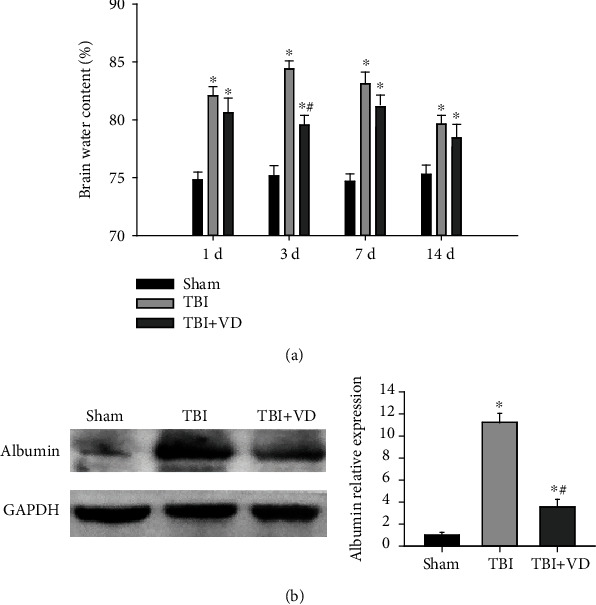
The assessment of brain water content and BBB permeability after TBI. (a) Brain water content was reduced in the TBI+VD group at days 3, 7, and 14 after TBI. (b) BBB dysfunction was estimated by analyzing albumin protein level in the hippocampus at day 3 post-TBI. The data are represented as mean ± SD from three independent experiments. ^∗^*P* < 0.05 compared with the sham group, ^#^*P* < 0.05 compared with the TBI group.

Brain edema, known as secondary brain injury, is an important and common pathophysiological feature following TBI. Herein, we measured brain water content using the wet weight-dry weight method at 1 d, 3 d, 7 d, and 14 d after TBI. The brain water content in the TBI group was significantly higher than that in the sham group (*P* < 0.05), which was obviously reduced in the TBI+VD group (*P* < 0.05, Figure 3(a)). The blood-brain barrier (BBB) disruption has been regarded as a major contributor to maintenance of chronic inflammation following TBI [25]. The level of albumin in the brain injury 3 d postinjury was detected using Western blot to assess BBB integrity. Data showed that hippocampal expression of albumin was evidently increased in the TBI group relative to the sham group (*P* < 0.05, Figure 3(b)). VD injection distinctly decreased albumin level after TBI (*P* < 0.05), indicating that VD efficiently improves BBB integrity following TBI in rats.

### 3.4. Vitamin D Contributed to Neuronal Survival In Vivo and In Vitro

**Figure 4:**
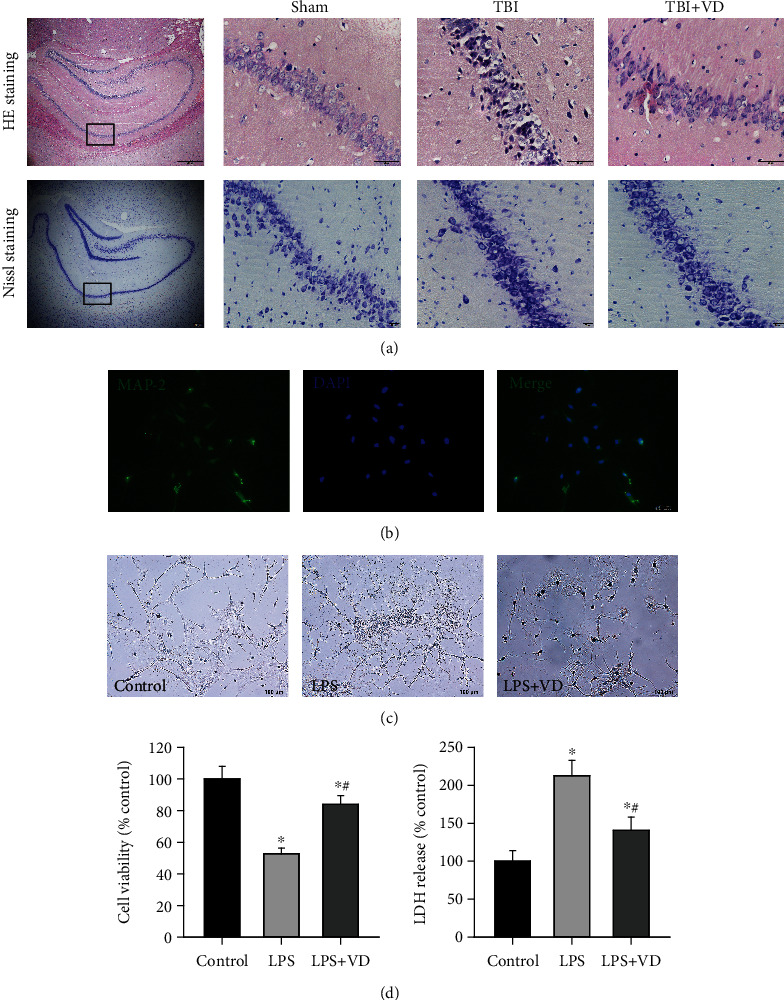
VD promoted hippocampal neuronal survival in vivo and in vitro. (a) H&E staining and Nissl staining of the hippocampal CA1 region in each group at day 3 post-TBI. Scale bar: 50 *μ*m and 20 *μ*m. (b) Immunofluorescent staining of the neuron biomarker MAP-2 in primary rat hippocampal neurons in vitro. Scale bar: 50 *μ*m. (c) Morphological changes of cultured neurons after LPS stimulation. (d) Neuronal viability and neuronal injury were evaluated by the MTT assay and LDH content. The data are represented as mean ± SD from three independent experiments. ^∗^*P* < 0.05 compared with the control group, ^#^*P* < 0.05 compared with the LPS group.

In the rat model of experimental TBI, perspective images of H&E and Nissl staining illustrated that healthy neurons in the sham group had a relatively large soma and round nuclei, together with a large amount of Nissl body in the cytoplasm. The damaged neurons shrank and contain a small amount of Nissl body in the TBI group; these neuronal injuries were significantly alleviated by the treatment with VD at 3 d posttrauma in vivo (Figure 4(a)). In in vitro studies, as shown in Figure 4(b), we identified cultured hippocampal neurons by immunofluorescent staining of the neuron-specific protein MAP-2. Using an LPS-induced neuron injury model, morphological images showed that VD might contribute to primary hippocampal neuronal survival after LPS stimulation (Figure 4(c)). The cytoprotective effects of VD were also confirmed by enhanced cellular viability (*P* < 0.05) and LDH reduction (*P* < 0.05, Figure 4(d)) in the culture supernatant of primary rat hippocampal neurons, using the MTT assay and LDH content measurement.

### 3.5. Vitamin D Inhibited Oxidative Stress and Neuroinflammation after TBI

**Figure 5:**
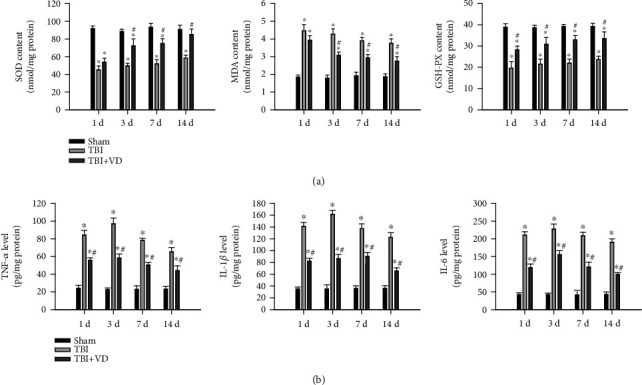
The roles of VD in oxidative stress and inflammatory response following TBI in rats. (a) Measurement of SOD, MDA, and GSH-PX activity in the hippocampal homogenate posttrauma. (b) The levels of proinflammation cytokines (TNF-*α*, IL-6, and IL-1*β*) were quantified by the ELISA method. The data are represented as mean ± SD from three independent experiments. ^∗^*P* < 0.05 compared with the control group, ^#^*P* < 0.05 compared with the LPS group.

Oxidative stress has been demonstrated to exacerbate neuroinflammatory response and neuronal cell death, presenting a key role in the pathogenesis of brain injury [26]. In comparison to the sham group, antioxidant enzymes SOD and GSH-PX are decreased (*P* < 0.05) and oxidative marker MDA activity is increased (*P* < 0.05) in the supernatant from the hippocampal homogenate (Figure 5(a)). Notably, VD might notably reverse these alterations of oxidative stress-related enzymes induced by TBI (*P* < 0.05). Besides, the concentrations of inflammatory mediators (TNF-*α*, IL-1*β*, and IL-6) in the hippocampal tissues were significantly higher in the TBI group than in the sham group (*P* < 0.05). The hippocampal expressions of inflammatory mediators in the rats of the TBI+VD group were downregulated compared with those of the TBI group (*P* < 0.05, Figure 5(b)). Altogether, these results suggested that VD supplement exhibited effectively antioxidative and anti-inflammatory activities in the experimental TBI model.

### 3.6. Vitamin D Repressed the TLR4/MyD88/NF-*κ*B Signaling Pathway

**Figure 6:**
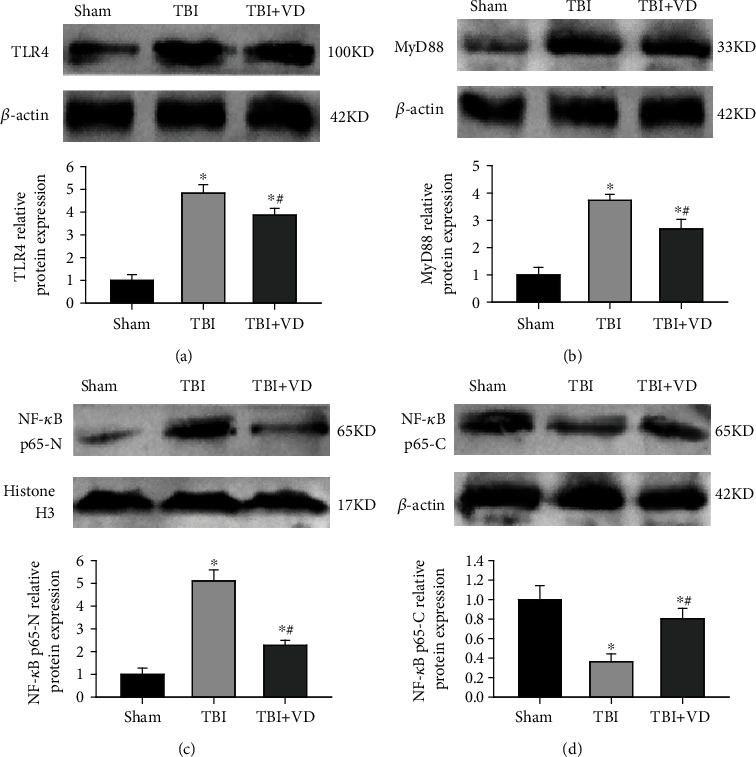
The effects of VD on the TLR4/MyD88/NF-*κ*B signaling pathway in the hippocampal tissue of rats. (a–d) Representative Western blots and densitometric quantification of hippocampal expression of TLR4, MyD88, nuclear NF-*κ*B p65, and cytoplasmic NF-*κ*B p65 protein at 3 d post-TBI in rats. The data are represented as mean ± SD from three independent experiments. ^∗^*P* < 0.05 compared with the sham group, ^#^*P* < 0.05 compared with the TBI group.

To investigate the molecular signal mechanisms in the involvement of VD-mediated neuroprotective effects in TBI rats, we detected toll-like receptor 4 (TLR4), myeloid differentiation factor 88 (MyD88), and NF-*κ*B p65 protein expression at 3 days post-TBI. TLR4 has been reported to mediate glial phagocytic activity and initiate the inflammatory response after TBI [27]. MyD88, as a critical adapter protein for TLR4, may contribute to the production of proinflammatory factors via activating the downstream NF-*κ*B signal [28]. Western blot results showed that hippocampal expression of TLR4, MyD88, and nuclear NF-*κ*B p65 protein was dramatically increased, whereas cytoplasmic NF-*κ*B p65 expression was decreased in the TBI group compared with the sham group (*P* < 0.05), but these changes were repressed by VD supplement (*P* < 0.05, Figures 6(a)–6(d)). The above data indicated that treatment with VD could lead to the inactivation of the TLR4/MyD88/NF-*κ*B signaling pathway in a rat model of TBI.

## 4. Discussion

Adequate VD level is essential for the maintenance of calcium homeostasis, skeletal integrity, neurodevelopment, and adult brain function [29]. Strong evidence in prospective observational studies identified that the VD deficiency (VDD) was associated with brain injury severity, neurocognitive disorder, depressive symptoms, and quality of life [30–32]. Besides, a retrospective cohort study found that VDD could increase probability of the occurrence of acute deep venous thrombosis in patients with moderate or severe TBI [33]. Accordingly, administration of VD supplements has been shown to improve long-term performance and cognitive outcomes and modulated immune function in TBI patients [20, 21, 34]. However, the detailed molecular mechanisms responsible for the clinical therapeutic effects of VD remain elusive. In this study, we first observed the neuroprotective role of VD supplement in the experimental TBI model. A rat model of TBI was established via CCI. CCI is the most widely used and investigated TBI model due to its flexibility in location and degree of injury and effectively mimics important neuropathological features of clinical TBI [35]. After the intraperitoneal injection of VD, the serum concentration of VD and the hippocampal expression of VDR protein were significantly increased at 3 d, 7 d, and 14 d post-CCI, highlighting an important role of VD in modulating hippocampus-dependent cognitive functions. Subsequently, our neurobehavioral data clearly validated that VD therapy could improve sensorimotor dysfunctions and long-term cognitive impairments in rats subjected to TBI, which is consistent with the clinical trials above. We also found that VD treatment markedly attenuated brain edema and BBB disruption, particularly reduce hippocampal neuronal cell loss in vivo, and contributed to neuronal survival in rat primary hippocampal neurons in vitro. Importantly, to explore the effects of VD treatment on excessive oxidative stress and inflammatory response following TBI in rats, we found that VD3 therapy significantly upregulated antioxidant enzyme activity and increased oxidative enzyme activity and proinflammatory mediator contents in the hippocampus following TBI. These results showed that VD obviously inhibited hippocampal oxidative stress and neuroinflammatory response in rats with TBI, subsequently leading to the improvement of tissue and neurological impairments.

Neuroinflammation involving microglial and astrocyte activation might contribute to neuronal damage and neurobehavioral deficits following TBI. Toll-like receptor 4 (TLR4), a member of the toll-like receptor family, plays a crucial role in inflammatory responses and innate immunity [28]. TLR4, extensively expressed in the brain, recognizes not only pathogen-associated molecular patterns (PAMP) but also damage-associated molecular patterns (DAMPs) to induce inflammatory cascade reactions in brains [36, 37]. Therefore, this signaling cascade has been proposed as an important therapeutic target in secondary brain injury posttrauma. Our previous study validated that mRNA and protein levels of endogenous TLR4 were markedly upregulated in the hippocampus in the early stage post-TBI [38]. Emerging evidence uncovered that the TLR4 pathway impaired synaptic plasticity and neurovascular integrity through microglial and astrocyte communication-mediated astrocyte activation posttrauma [39]. Additionally, a significant correlation between TLR4 expressed on the surface of the neural stem cell (NSCs) and NSC proliferation/differentiation was monitored in mouse hippocampal tissues after TBI [40]. Nevertheless, inhibition of TLR4 by electroacupuncture could improve hippocampal neurogenesis in a mouse model of TBI [41]. Our previous study also revealed that inhibition of TLR4 restored posttraumatic histological and functional outcomes through repressing hippocampal neuronal autophagy and astrocyte activation after TBI [38]. In the present study, we investigated the effects of VD supplementation on TLR4 protein and its downstream cascade signal in a rat model of TBI. As an important transmembrane receptor, TLR4 is able to primarily activate the downstream NF-*κ*B signal to induce the release of proinflammatory cytokines and chemokine genes via recruiting adapter protein MyD88 in response to brain injury. Herein, Western blot analysis showed that TBI-induced activation of the TLR4/MyD88/NF-*κ*B signaling pathway was suppressed by VD administration.

## 5. Conclusion

Taken together, these findings clearly indicated that VD therapy promoted microglial polarization toward the M2 phenotype and subsequent neuroinflammation through suppressing the TLR4/MyD88/NF-*κ*B signaling pathway following TBI, finally contributing to the restoration of posttraumatic secondary brain injury in rats. Therefore, this study will provide a promising therapeutic strategy for TBI in the future.

## Data Availability

The data used to support the findings of this study are available from the corresponding author upon request.

## Supplementary Materials

Supplementary Materials Supplementary Data 1: VD induced the switch of microglia toward the M2 phenotype via the TLR4 pathway. (A) The mRNA levels of proinflammatory cytokines and M1 microglial marker were monitored by real-time PCR at 3 d post-TBI. (B) The mRNA levels of anti-inflammatory mediators and M2 microglial marker were monitored by real-time PCR at 3 d postinjury. (C) Representative Western blots and densitometric quantification of M1 and M2 marker (iNOS and Arg-1) expression in the hippocampus. The data are represented as mean ± SD from three independent experiments. ^∗^*P* < 0.05 compared with the sham group, ^#^*P* < 0.05 compared with the TBI group, and ^&^*P* < 0.05 compared with the TBI+VD group.

## References

[1] Cui X., Gooch H., Petty A., McGrath J. J., Eyles D. (2017). Vitamin D and the brain: genomic and non-genomic actions. *Molecular and Cellular Endocrinology*.

[2] Piotrowska A., Wierzbicka J., Żmijewski M. A. (2016). Vitamin D in the skin physiology and pathology. *Acta Biochimica Polonica*.

[3] Mayne P. E., Burne T. H. J. (2019). Vitamin D in synaptic plasticity, cognitive function, and neuropsychiatric illness. *Trends in Neurosciences*.

[4] Bivona G., Gambino C. M., Iacolino G., Ciaccio M. (2019). Vitamin D and the nervous system. *Neurological Research*.

[5] DeLuca G. C., Kimball S. M., Kolasinski J., Ramagopalan S. V., Ebers G. C. (2013). Review: the role of vitamin D in nervous system health and disease. *Neuropathology and Applied Neurobiology*.

[6] Eyles D. W., Smith S., Kinobe R., Hewison M., McGrath J. J. (2005). Distribution of the vitamin D receptor and 1*α*-hydroxylase in human brain. *Journal of Chemical Neuroanatomy*.

[7] Tuoresmäki P., Väisänen S., Neme A., Heikkinen S., Carlberg C. (2014). Patterns of genome-wide VDR locations. *PLoS One*.

[8] Pike J. W., Lee S. M., Meyer M. B. (2014). Regulation of gene expression by 1, 25-dihydroxyvitamin D3 in bone cells: exploiting new approaches and defining new mechanisms. *BoneKEy Reports*.

[9] Meyer M. B., Goetsch P. D., Pike J. W. (2010). A downstream intergenic cluster of regulatory enhancers contributes to the induction of CYP24A1 expression by 1alpha, 25-dihydroxyvitamin D3. *The Journal of Biological Chemistry*.

[10] Ashouri R., Fangman M., Brielmaier J., Fields Z. A., Campo N., Doré S. (2021). Nutritional supplementation of naturally occurring vitamin D to improve hemorrhagic stroke outcomes. *Frontiers in Neurology*.

[11] Albiñana C., Boelt S. G., Cohen A. S. (2021). Developmental exposure to vitamin D deficiency and subsequent risk of schizophrenia. *Schizophrenia Research*.

[12] Aspell N., Lawlor B., O'Sullivan M. (2018). Is there a role for vitamin D in supporting cognitive function as we age?. *The Proceedings of the Nutrition Society*.

[13] Lazzara F., Amato R., Platania C. B. M. (2021). 1*α*, 25-dihydroxyvitamin D3 protects retinal ganglion cells in glaucomatous mice. *Journal of Neuroinflammation*.

[14] Ma J. G., Wu G. J., Xiao H. L., Xiao Y. M., Zha L. (2021). Vitamin D has an effect on airway inflammation and Th17/Treg balance in asthmatic mice. *The Kaohsiung Journal of Medical Sciences*.

[15] Dinet V., Petry K. G., Badaut J. (2019). Brain-immune interactions and neuroinflammation after traumatic brain injury. *Frontiers in Neuroscience*.

[16] Dixon K. J. (2017). Pathophysiology of traumatic brain injury. *Physical Medicine and Rehabilitation Clinics of North America*.

[17] Mishra A., Bandopadhyay R., Singh P. K., Mishra P. S., Sharma N., Khurana N. (2021). Neuroinflammation in neurological disorders: pharmacotherapeutic targets from bench to bedside. *Metabolic Brain Disease*.

[18] Sharma S., Kumar A., Choudhary A. (2020). Neuroprotective role of oral vitamin D supplementation on consciousness and inflammatory biomarkers in determining severity outcome in acute traumatic brain injury patients: a double-blind randomized clinical trial. *Clinical Drug Investigation*.

[19] Zhang Y., Wu Y., Guo J. (2020). Correlation between vitamin D and cognitive function in patients with traumatic brain injury in China. *Applied Neuropsychology. Adult*.

[20] Lee J. M., Jeong S. W., Kim M. Y., Park J. B., Kim M. S. (2019). The effect of vitamin D supplementation in patients with acute traumatic brain injury. *World Neurosurgery*.

[21] Choudhary A., Kumar A., Sharma R. (2021). Optimal vitamin D level ameliorates neurological outcome and quality of life after traumatic brain injury: a clinical perspective. *The International Journal of Neuroscience*.

[22] Yang J., Wang K., Hu T., Wang G., Wang W., Zhang J. (2021). Vitamin D3 supplement attenuates blood-brain barrier disruption and cognitive impairments in a rat model of traumatic brain injury. *Neuromolecular Medicine*.

[23] Dixon C. E., Clifton G. L., Lighthall J. W., Yaghmai A. A., Hayes R. L. (1991). A controlled cortical impact model of traumatic brain injury in the rat. *Journal of Neuroscience Methods*.

[24] Hollis B. W. (1996). Assessment of vitamin D nutritional and hormonal status: what to measure and how to do it. *Calcified Tissue International*.

[25] van Vliet E. A., Ndode-Ekane X. E., Lehto L. J. (2020). Long-lasting blood-brain barrier dysfunction and neuroinflammation after traumatic brain injury. *Neurobiology of Disease*.

[26] Lan Y. L., Zhu Y., Chen G., Zhang J. (2021). The promoting effect of traumatic brain injury on the incidence and progression of glioma: a review of clinical and experimental research. *Journal of Inflammation Research*.

[27] Ahmad A., Crupi R., Campolo M., Genovese T., Esposito E., Cuzzocrea S. (2013). Absence of TLR4 reduces neurovascular unit and secondary inflammatory process after traumatic brain injury in mice. *PLoS One*.

[28] Rahimifard M., Maqbool F., Moeini-Nodeh S. (2017). Targeting the TLR4 signaling pathway by polyphenols: a novel therapeutic strategy for neuroinflammation. *Ageing Research Reviews*.

[29] Anjum I., Jaffery S. S., Fayyaz M., Samoo Z., Anjum S. (2018). The role of vitamin D in brain health: a mini literature review. *Cureus*.

[30] Toman E., Bishop J. R., Davies D. J. (2017). Vitamin D deficiency in traumatic brain injury and its relationship with severity of injury and quality of life: a prospective, observational study. *Observational Study. J Neurotrauma.*.

[31] Jamall O. A., Feeney C., Zaw-Linn J. (2016). Prevalence and correlates of vitamin D deficiency in adults after traumatic brain injury. *Clinical Endocrinology*.

[32] Dubiel R., Williams B., Sullivan E., Callender L., Bennett M., Driver S. (2019). Prevalence of 25-hydroxyvitamin D deficiency in the acute rehabilitation population following traumatic brain injury. *NeuroRehabilitation*.

[33] Moore M., Goldin Y., Patel H., Greenwald B. D. (2021). Low vitamin D level is associated with acute deep venous thrombosis in patients with traumatic brain injury. *Brain Sciences*.

[34] Arabi S. M., Sedaghat A., Ehsaei M. R. (2020). Efficacy of high-dose versus low-dose vitamin D supplementation on serum levels of inflammatory factors and mortality rate in severe traumatic brain injury patients: study protocol for a randomized placebo-controlled trial. *Trials*.

[35] Dean D. D., Frank J. A., Turtzo L. C. (2017). Controlled cortical impact in the rat. *Current Protocols in Neuroscience*.

[36] Carty M., Bowie A. G. (2011). Evaluating the role of Toll-like receptors in diseases of the central nervous system. *Biochemical Pharmacology*.

[37] Gárate I., García-Bueno B., Madrigal J. L. (2014). Toll-like 4 receptor inhibitor TAK-242 decreases neuroinflammation in rat brain frontal cortex after stress. *Journal of Neuroinflammation*.

[38] Jiang H., Wang Y., Liang X., Xing X., Xu X., Zhou C. (2018). Toll-like receptor 4 knockdown attenuates brain damage and neuroinflammation after traumatic brain injury via inhibiting neuronal autophagy and astrocyte activation. *Cellular and Molecular Neurobiology*.

[39] Rosa J. M., Farré-Alins V., Ortega M. C. (2021). TLR4 pathway impairs synaptic number and cerebrovascular functions through astrocyte activation following traumatic brain injury. *British Journal of Pharmacology*.

[40] Ye Y., Xu H., Zhang X. (2014). Association between toll-like receptor 4 expression and neural stem cell proliferation in the hippocampus following traumatic brain injury in mice. *International Journal of Molecular Sciences*.

[41] Ye Y., Yang Y., Chen C. (2017). Electroacupuncture improved hippocampal neurogenesis following traumatic brain injury in mice through inhibition of TLR4 signaling pathway. *Stem Cells International*.

