# Supplementary figures and images for: Vitamin D Protects against Traumatic Brain Injury via Modulating TLR4/MyD88/NF-κB Pathway-Mediated Microglial Polarization and Neuroinflammation
Source: Biomed Res Int. 2022 Jul 15;2022:3363036. doi: 10.1155/2022/3363036 (PMC9307360; doi:10.1155/2022/3363036)

**A**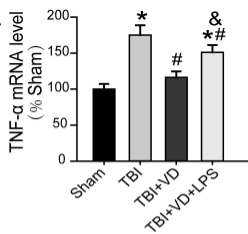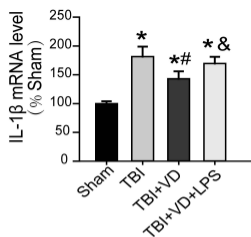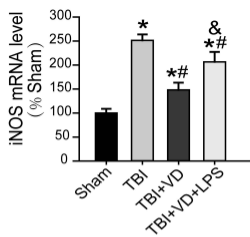**B**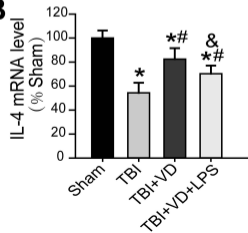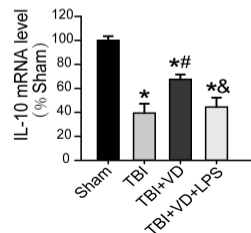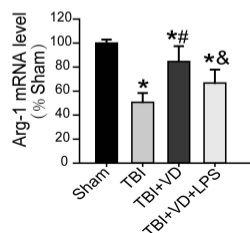**C**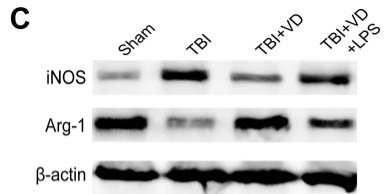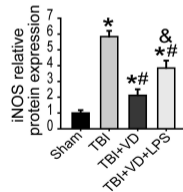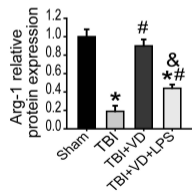

Supplement: Supplementary Materials — Supplementary Data 1: VD induced the switch of microglia toward the M2 phenotype via the TLR4 pathway. (A) The mRNA levels of proinflammatory cytokines and M1 microglial marker were monitored by real-time PCR at 3 d post-TBI. (B) The mRNA levels of anti-inflammatory mediators and M2 microglial marker were monitored by real-time PCR at 3 d postinjury. (C) Representative Western blots and densitometric quantification of M1 and M2 marker (iNOS and Arg-1) expression in the hippocampus. The data are represented as mean ± SD from three independent experiments. ∗P < 0.05 compared with the sham group, #P < 0.05 compared with the TBI group, and &P < 0.05 compared with the TBI+VD group. [file 3363036.f1.pdf]
